# Possible superconductivity in Sr_2_IrO_4_ probed by quasiparticle interference

**DOI:** 10.1038/srep09251

**Published:** 2015-03-18

**Authors:** Yi Gao, Tao Zhou, Huaixiang Huang, Qiang-Hua Wang

**Affiliations:** 1 Department of Physics and Institute of Theoretical Physics, Nanjing Normal University, Nanjing. 210023, China; 2 College of Science, Nanjing University of Aeronautics and Astronautics, Nanjing. 210016, China; 3 Department of Physics, Shanghai University, Shanghai. 200444, China; 4 National Laboratory of Solid State Microstructures, Nanjing University, Nanjing. 210093, China

## Abstract

Based on the possible superconducting (SC) pairing symmetries recently proposed, the quasiparticle interference (QPI) patterns in electron- and hole-doped Sr_2_IrO_4_ are theoretically investigated. In the electron-doped case, the QPI spectra can be explained based on a model similar to the octet model of the cuprates while in the hole-doped case, both the Fermi surface topology and the sign of the SC order parameter resemble those of the iron pnictides and there exists a QPI vector resulting from the interpocket scattering between the electron and hole pockets. In both cases, the evolution of the QPI vectors with energy and their behaviors in the nonmagnetic and magnetic impurity scattering cases can well be explained based on the evolution of the constant-energy contours and the sign structure of the SC order parameter. The QPI spectra presented in this paper can be compared with future scanning tunneling microscopy experiments to test whether there are SC phases in electron- and hole-doped Sr_2_IrO_4_ and what the pairing symmetry is.

Recently, a very interesting material, the 5*d* transition metal oxide Sr_2_IrO_4_ has attracted much attention^1^,^2^,^3^,^4^,^5^,^6^,^7^,^8^,^9^,^10^,^11^,^12^,^13^,^14^. In this material, the energy bands close to the Fermi level are mainly contributed by the *t*_2*g*_ orbitals of Ir and it is in the (*t*_2*g*_)^5^ configuration. On the one hand, due to the extended nature of 5*d* orbitals, Coulomb interaction *U* for 5*d* electrons (1–3 eV) is expected to be smaller than that for 3*d* electrons (5–7 eV)^7^. On the other hand, the spin-orbit coupling (SOC) is considerably larger by a factor of 10 in 5*d* than in 3*d*^7^. In this case, the strong SOC splits the *t*_2*g*_ orbitals into an upper *J* = 1/2 band and lower *J* = 3/2 bands. In the parent compound, the *J* = 3/2 bands are fully occupied while the *J* = 1/2 band is half-filled. Meanwhile, the bandwidth of this *J* = 1/2 band is much smaller than the original one in the absence of the SOC. Therefore, even a small *U* can lead the system into a Mott insulator with pseudospin 1/2 antiferromagnetic (AFM) order, making Sr_2_IrO_4_ an analog to the parent compound of the cuprates. This *J* = 1/2 AFM Mott insulating state is supported by several experiments^4^,^5^,^6^,^8^,^10^,^11^,^12^,^13^. Therefore, Sr_2_IrO_4_ is an ideal candidate to perform comparative studies with the cuprates. The question is, whether doping Sr_2_IrO_4_ can induce superconductivity in analogy to the cuprates?

To resolve this issue, Refs. 15 and 16 theoretically investigated the superconducting (SC) properties in both electron- and hole-doped Sr_2_IrO_4_. They found that, in the electron-doped case, a SC phase indeed exists and the pairing contains both intraorbital and interorbital components as well as both singlet and triplet components of *t*_2*g*_ electrons, while the pairing symmetry on the Fermi surface is 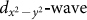 (or 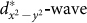 as denoted by Ref. 16) and the pairing function respects time-reversal symmetry (TRS), similar to the cuprates. On the other hand, in the hole-doped case, the Fermi surface topology changes and resembles that of the iron pnictides, with an electron pocket around the Γ point and a hole pocket around the *M* point. In this case, Ref. 15 found that there is no SC phase while Ref. 16 concluded that a SC phase can also exist while the pairing function still respects TRS and the pairing symmetry is 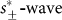, similar to that of the iron pnictides^17^.

In this paper, in order to search for an experimental test of the above two theories, we propose to measure the quasiparticle interference (QPI) patterns in both electron- and hole-doped Sr_2_IrO_4_ by scanning tunneling microscopy (STM). As we know, the QPI patterns are strongly influenced by the shape and evolution of the constant-energy contour (CEC), as well as the relative sign of the SC order parameter of the states connected by the QPI wave vectors^18^,^19^,^20^,^21^,^22^. Therefore, by measuring the QPI patterns, we can not only determine whether the SC phase exists in the electron- and hole-doped cases, but also the SC pairing symmetry.

## Methods

We start with the lattice model adopted in Refs. 15 and 16, which takes the three *t*_2*g*_ orbitals (*d_xz_*, *d_yz_* and *d_xy_*) of Ir into account. The Hamiltonian can be written as
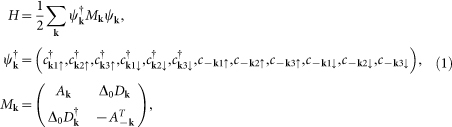
where
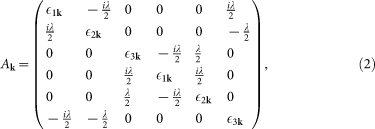
and
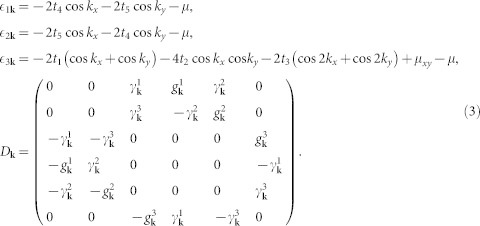
Here 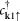, 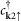 and 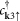 create a spin-up electron with momentum **k** in the *d_xz_*, *d_yz_* and *d_xy_* orbitals, respectively. *A***_k_** stands for the tight-binding part of the Hamiltonian in the presence of the SOC, with *λ* being the SOC strength. (*t*_1_, *t*_2_, *t*_3_, *t*_4_, *t*_5_, *μ_xy_*, *λ*) = (0.36, 0.18, 0.09, 0.37, 0.06, −0.36, 0.5) and *μ* is the chemical potential which is adjusted according to the electron filling *n*. *D***_k_** describes the pairing term of the Hamiltonian whose explicit expression is given later and we set Δ_0_ = 0.05 (unless otherwise specified).

When a single impurity is located at the origin, the impurity Hamiltonian can be written as
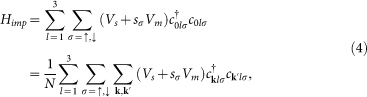
with *N* being the system size (396 × 396 throughout the paper) and *s_σ_* = 1(−1) for *σ* = ↑ (↓). We consider both nonmagnetic and magnetic impurity scattering, diagonal in the orbital basis and with a scattering strength *V_s_* and *V_m_* for the nonmagnetic and magnetic cases, respectively. For definiteness, *V_s_* and *V_m_* are both taken to be 0.04. Following the standard *T*-matrix procedure^23^, the Green's function matrix is defined as
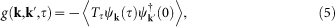
and
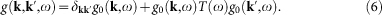
Here *g*_0_(**k**, *ω*) is the Green's function in the absence of the impurity and can be written as
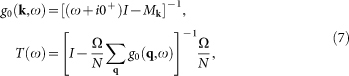
where *I* is a 12 × 12 unit matrix and
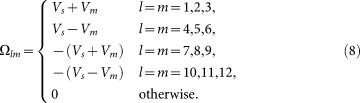
The experimentally measured local density of states (LDOS) is expressed as
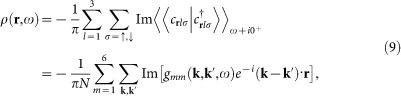
and its Fourier transform is defined as 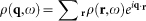. Since the system is even under **k** → −**k** (*D***_k_** is also an even function of **k** as can be seen later), it can be written as
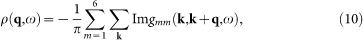
and the contribution from the spin up and spin down electrons can be expressed as
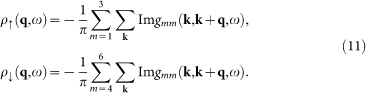


Here we need to clarify what to measure in the STM experiment. If the impurity scattering is weak, then *T*(*ω*) ∝ Ω. In this case, if *V_s_* ≠ 0 and *V_m_* = 0, we have *ρ*_↑_(**q**, *θ*) = *ρ*_↓_(**q**, *ω*) since the system respects TRS. On the other hand, if *V_s_* = 0 and *V_m_* ≠ 0, TRS is broken and now for **q** ≠ 0, we have *ρ*_↑_(**q**, *ω*) = −*ρ*_↓_(**q**, *ω*), leading to *ρ*(**q**, *ω*) = 0. Therefore, in the STM experiment, people should measure the spin-resolved LDOS, either *ρ*_↑_(**r**, *ω*) or *ρ*_↓_(**r**, *ω*), to get a nontrivial QPI spectrum.

## Results and discussion

**Figure 1:**
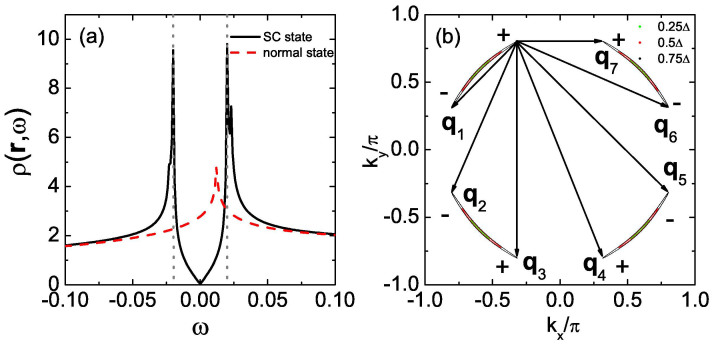
At *n* = 5.2. (a) *ρ*(**r**, *ω*) as a function of *ω*, in the absence of the impurity. The gray dotted lines denote the position of the two SC coherence peaks, located at ±Δ (Δ ≈ 0.4Δ_0_). (b) The CEC at |*ω*|/Δ = 0.25 (green), 0.5 (red) and 0.75 (black). **q**_1_, **q**_2_, …, **q**_7_ are characteristic QPI wave vectors connecting the tips of the CEC. The + and − denote the sign of the SC order parameter on the CEC.

At *n* = 5.2, the electron-doped case, the pairing functions *g***_k_** and *γ***_k_** in equation (3) can be expressed as^16^
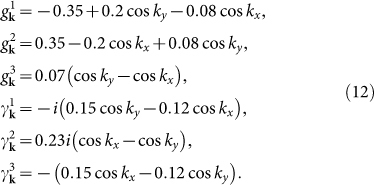
The LDOS in the absence of the impurity is homogeneous in real space and is shown in Fig. 1(a). Two SC coherence peaks are located at ±Δ where Δ ≈ 0.4Δ_0_ and the spectrum is V-shaped in the vicinity of *ω* = 0, indicating the nodal gap structure, consistent with the 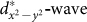 pairing symmetry.

**Figure 2:**
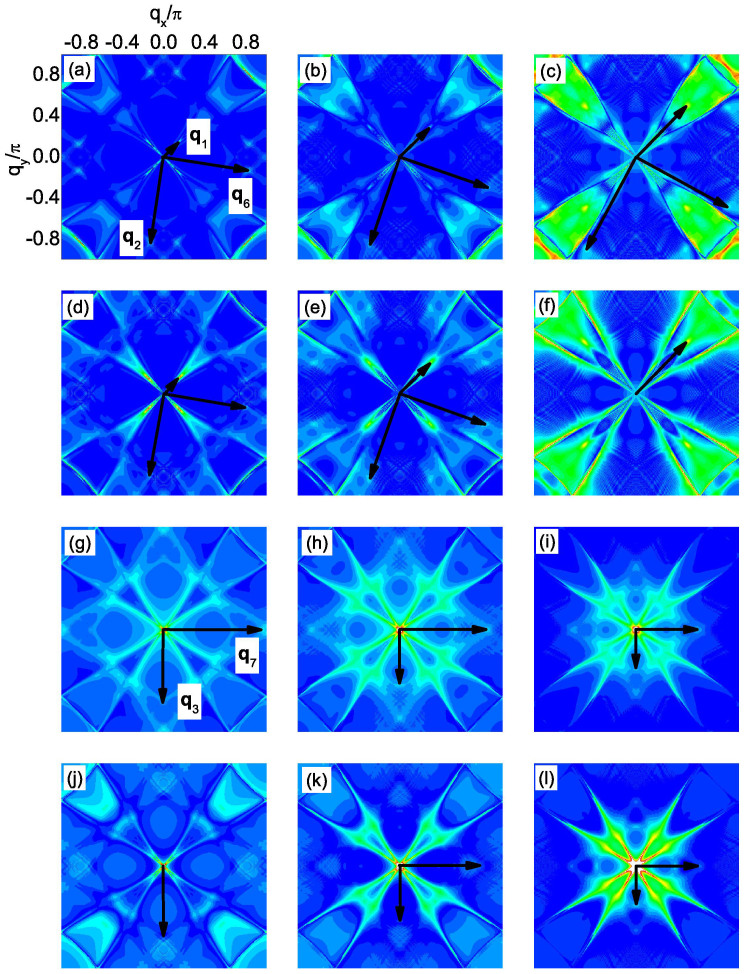
At *n* = 5.2, |*ρ*_↑_(q, *ω*)| at fixed *ω*. The point at **q** = 0 is neglected in order to show weaker features at other wave vectors. (a–f) *ω*/Δ = −0.25, −0.5, −0.75, 0.25, 0.5, 0.75, for the nonmagnetic impurity scattering. (g–l) are the same as (a–f), but for the magnetic impurity scattering.

In the presence of the impurity, we plot |*ρ*_↑_(**q**, *ω*)| in Fig. 2 and several QPI wave vectors can be identified. For nonmagnetic impurity scattering [from Figs. 2(a) to 2(f)], three QPI wave vectors **q**_1_, **q**_2_ and **q**_6_ can be clearly seen evolving with energy. **q**_1_ is located along the (±1, ±1) directions and moves away from the origin as |*ω*| increases. **q**_2_ and **q**_6_ are not located along the high-symmetry directions and they overlap after a 90 degree rotation. Furthermore, they are not so obvious at *ω*/Δ = 0.75 since they are masked by the high-intensity spots around them. In contrast, for magnetic impurity scattering [from Figs. 2(g) to 2(l)], **q**_1_, **q**_2_ and **q**_6_ become less clear and instead, another two vectors **q**_3_ and **q**_7_ can be identified evolving with energy. They are both located along the (0, ±1) and (±1, 0) directions and move towards the origin as |*ω*| increases.

The appearance and evolution of the above five QPI wave vectors can be understood from the evolution of the CEC. As we can see from Fig. 1(b), the CEC of the electron-doped Sr_2_IrO_4_ is similar to the octet model of the cuprates^18^,^19^,^20^,^21^,^23^ and the expected QPI vectors should be those connecting the tips of the CEC, *i.e.*, **q**_1_, **q**_2_, …, **q**_7_ in this case. For example, at |*ω*|/Δ = 0.5, **q**_1_, **q**_2_, …, **q**_7_ shown in Fig. 1(b) are located at (−0.295, −0.295), (−0.295, 0.839), (0, 0.544), (0.866, 0.544), (−0.839, 0.839), (−0.839, −0.295) and (0.866, 0), agree quite well with those shown in Figs. 2(b), 2(e), 2(h) and 2(k), except that **q**_4_ and **q**_5_ cannot be identified. At |*ω*|/Δ = 0.25 and 0.75, the locations of the QPI vectors shown in Fig. 1(b) are also consistent with those in Fig. 2 and for all the energies we investigated, **q**_4_ and **q**_5_ cannot be clearly seen, thus we neglect these two vectors in the following.

Next we discuss the implication of the QPI vectors on the sign of the SC order parameter. As we know, due to the effect of the SC coherence factors, those scattering between the states with the opposite (same) sign of the SC order parameters will be enhanced (suppressed) by nonmagnetic impurity. For magnetic impurity scattering, the situation is reversed. In electron-doped Sr_2_IrO_4_, since the pairing symmetry is assumed to be 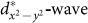 and the sign of the SC order parameter on the CEC is shown in Fig. 1(b) as + and −. As we can see, **q**_1_, **q**_2_ and **q**_6_ are sign-reversing scattering processes while **q**_3_ and **q**_7_ are sign-preserving ones. Therefore, **q**_1_, **q**_2_ and **q**_6_ should be more discernable in the nonmagnetic impurity scattering case while **q**_3_ and **q**_7_ should be more distinct in the magnetic impurity scattering case. This is exactly what we obtain here as can be seen from Fig. 2. Therefore, the evolution of the QPI vectors with energy together with their different behaviors in the nonmagnetic and magnetic impurity scattering cases can help to determine whether the pairing symmetry is 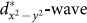 in electron-doped Sr_2_IrO_4_.

Here we need to point out that, Ref. 15 assumed that the SC pairing is a pseudospin singlet formed by the *J* = 1/2 Kramers doublet and the pairing symmetry is 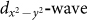. In this case, the pairing term of the Hamiltonian can be written as 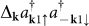, where 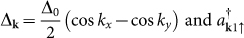 and 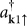 creates a pseudospin up electron with momentum **k** in the *J* = 1/2 band. If we set Δ_0_ = 0.02 here, then the LDOS in the absence of the impurity is qualitatively the same as that shown in Fig. 1(a) and now we have Δ = Δ_0_. In addition, the evolution of the CEC and the QPI spectra obtained are also similar to those in Figs. 1(b) and 2, respectively, indicating that the pairing functions adopted in Refs. 15 and 16 share the same characteristics. As we can see in the limit of large SOC (*λ* → ∞), 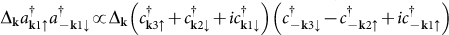. Although the pairing is a pseudospin singlet, it contains both intraorbital and interorbital components as well as both singlet and triplet components of *t*_2*g*_ electrons and it respects the same symmetry as that shown in equation (12). Therefore, for electron-doped Sr_2_IrO_4_, Refs. 15 and 16 predicted similar SC phases.

**Figure 3:**
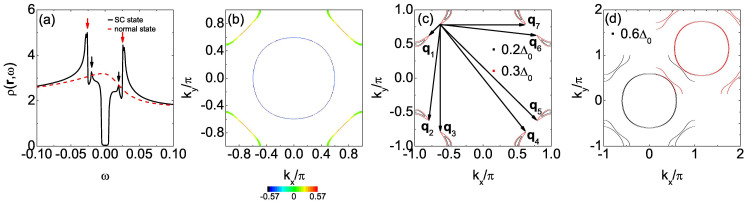
At *n* = 4.25. (a) The same as Fig. 1(a). (b) The pairing function *D***_k_** projected onto the Fermi surface. (c) The CECs at |*ω*| = 0.2Δ_0_ (black) and 0.3Δ_0_ (red). (d) The CEC at |*ω*| = 0.6Δ_0_. The red curves in (d) are displaced by (1.15*π*, 1.15*π*) from the black ones.

Then we consider the hole-doped Sr_2_IrO_4_ at *n* = 4.25. In this case, the pairing function proposed by Ref. 16 can be written as
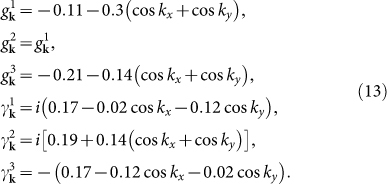
The LDOS in the absence of the impurity is shown in Fig. 3(a) and two pairs of SC coherence peaks are located at ±0.4Δ_0_ and ±0.52Δ_0_, as denoted by the black and red arrows, respectively, with a U-shaped profile close to *ω* = 0, indicating the full gap opening at this doping level. The pairing function *D***_k_** projected onto the Fermi surface is shown in Fig. 3(b). As we can see, the pairing order parameter on the electron pocket around Γ is negative and almost isotropic while on the hole pocket around *M*, it is positive and anisotropic. Therefore, the Fermi surface topology and the sign change of the pairing order parameter between the electron and hole pockets are very similar to the iron pnictides^17^,^22^ and this pairing symmetry is dubbed as 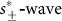.

The evolution of the CEC with energy at this doping level is shown in Figs. 3(c) and 3(d). As can be seen, at low energies (|*ω*| = 0.2Δ_0_ and 0.3Δ_0_), the CEC exists around the *M* point and the characteristic QPI vectors should be **q**_1_, **q**_2_, …, **q**_7_ as shown in Fig. 3(c). **q**_1_ and **q**_5_ are located along the (±1, ±1) directions. **q**_1_ moves towards the origin as |*ω*| increases while **q**_5_ hardly evolves with energy. **q**_2_ and **q**_6_ are not located along the high-symmetry directions while **q**_3_ and **q**_7_ are both located along the (0, ±1) and (±1, 0) directions. In addition, **q**_3_ should move towards the origin with increasing |*ω*| while the situation for **q**_7_ is reversed. As |*ω*| increases to 0.4Δ_0_, the tips of the two adjacent CECs touch each other. So in this case, **q**_1_ should disappear while **q**_2_, **q**_3_, **q**_6_ and **q**_7_ become equivalent. When |*ω*| ≥ 0.5Δ_0_, the CEC around *M* evolves into closed contours where no tips exist, thus the above mentioned QPI vectors disappear. Here **q**_1_, **q**_2_, …, **q**_7_ are all sign-preserving scattering processes, therefore they should be more discernable in the magnetic impurity scattering case. As |*ω*| increases to 0.6Δ_0_, another CEC shows up around the Γ point. In this case, a large portion of the CECs around the *M* and Γ points are quasinested with each other by a nesting vector (±1.15*π*, ±1.15*π*), as can be seen from Fig. 3(d). In this case, there should exist a QPI vector located at around (±0.85*π*, ±0.85*π*) in the first Brillouin zone and since it is a sign-reversing scattering process, it should be more distinct in the nonmagnetic impurity scattering case^22^.

**Figure 4:**
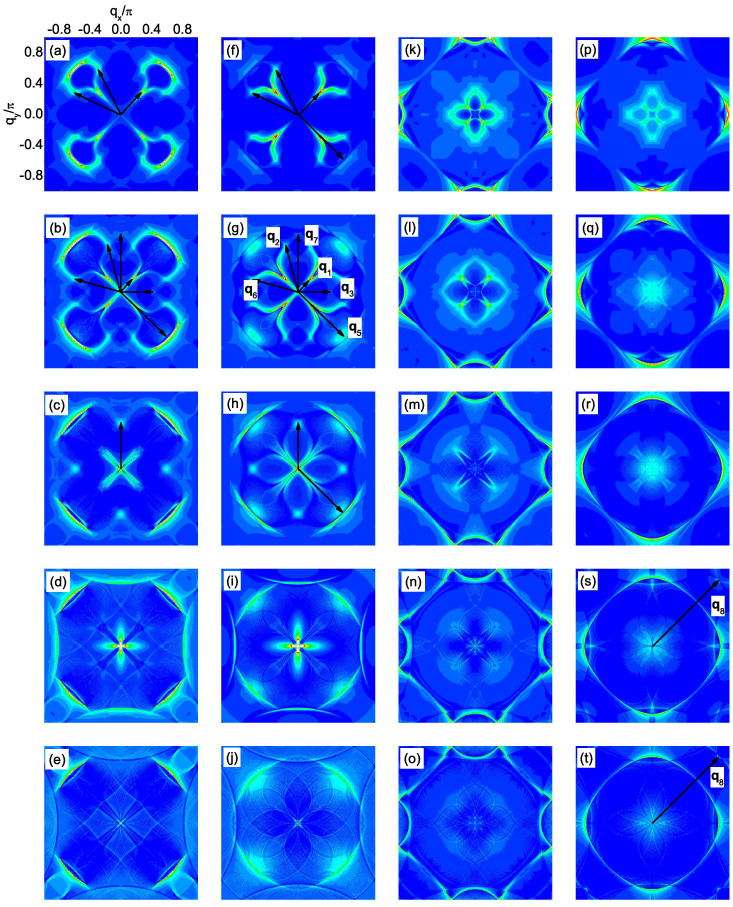
At *n* = 4.25, |*ρ*_↑_(q, *ω*)| at fixed *ω*. The point at **q** = 0 is neglected. (a–j): magnetic impurity scattering. (k–t): nonmagnetic impurity scattering. (a–e) and (k–o): *ω*/Δ_0_ = −0.2, −0.3, −0.4, −0.5, −0.6. (f–j) and (p–t): *ω*/Δ_0_ = 0.2, 0.3, 0.4, 0.5, 0.6.

To verify the above expectations, the QPI spectra are calculated and are plotted in Fig. 4. For magnetic impurity scattering [see Figs. 4(a) to 4(j)], indeed we can identify the QPI vectors **q**_1_, **q**_2_, …, **q**_7_, except that **q**_4_ cannot be clearly seen. The evolution of these vectors is also consistent with that derived from Fig. 3(c), *i.e.*, **q**_1_ locates along the (±1, ±1) directions and moves towards the origin with increasing |*ω*|. **q**_3_ and **q**_7_ both locate along the (0, ±1) and (±1, 0) directions while they become equivalent with **q**_2_ and **q**_6_ at |*ω*|/Δ_0_ = 0.4. Meanwhile, **q**_5_ barely evolves with energy and at |*ω*|/Δ_0_ ≥ 0.5, the above mentioned QPI vectors disappear. On the other hand, for nonmagnetic impurity scattering, as we can see from Figs. 4(k) to 4(t), **q**_1_, **q**_2_, …, **q**_7_ become less clear and instead, at *ω*/Δ_0_ = 0.5 and 0.6 [see Figs. 4(s) and 4(t)], another QPI vector **q**_8_ shows up at around (±0.85*π*, ±0.85*π*), which is resulted from the interpocket scattering between the electron and hole pockets as we mentioned above. Therefore, the locations of these QPI vectors and their behaviors in the nonmagnetic and magnetic impurity scattering cases are consistent with what we expected from the evolution of the CEC and the sign structure of the SC order parameter.

**Figure 5:**
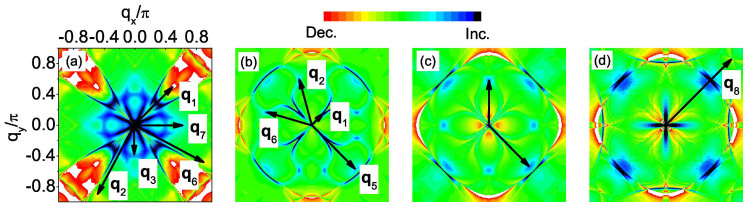
Δ|*ρ*_↑_(q, *ω*)|. (a): *n* = 5.2 and *ω*/Δ = −0.75. (b), (c) and (d) are all for *n* = 4.25, but at *ω*/Δ_0_ = 0.3, 0.4 and 0.5, respectively.

Here we need to point out that, in real STM experiments, both nonmagnetic and magnetic scatterers inevitably coexist in the same sample and are difficult to control. In this case, a magnetic field is usually applied to introduce additional scatters into the system. When an external magnetic field is applied, the main effects are the formation of vortices and the Zeeman splitting. In the following, we discuss these two effects separately. As pointed out in Refs. 21 and 22, the introduction of vortices causes the phase of the SC gap to precess by 2*π* around each vortex, whereas the amplitude of the gap vanishes at its core. Both the phase gradient and the inhomogeneity in the SC gap amplitude can scatter quasiparticles. The inhomogeneous superflow about the vortex (resulting from the phase gradient) produces Doppler-shift scattering that is odd under time reversal like magnetic impurities, while the spatial inhomogeneity in the SC gap amplitude causes inhomogeneous Andreev scattering. Although the vortex core is not a simple magnetic impurity as shown in equation (4), all of these scatterings selectively activate the sign-preserving **q** points. Especially, from Table S1 in the supporting online material for Ref. 21 we can see clearly that both the phase gradient and the gap amplitude scatterings enhance the same **q***_i_* (*i* = 1, 4, 5) as the magnetic impurity does. This is further confirmed by Maltseva and Coleman^24^ who found that both the Andreev scattering and the resonant scattering (whose coherence factors are the same as those of the phase-gradient scattering) are equally effective in qualitatively modeling the observations, *i.e.*, they both enhance the same **q***_i_* (*i* = 1, 4, 5) as the magnetic impurity does. On the other hand, strictly speaking, in order to study the effect of vortices on the QPI, we should solve the Bogoliubov-de Gennes (BdG) equations in real space to get the phase and amplitude variation of the SC gap self-consistently by introducing a Peierls phase factor in the hopping integral *t*. However in our work, the pairing function is given in momentum space and it is very difficult to use a real-space attraction to simulate this momentum-space pairing function. Therefore it is impossible for us to solve the real-space BdG equations to exactly investigate the effects of vortices on the QPI. Thus in our work, the scattering off vortices can be approximated as the scattering off the magnetic impurity. In Fig. 5 we show the difference of the QPI spectra between the magnetic and nonmagnetic impurity scattering cases, defined as Δ|*ρ*_↑_(**q**, *ω*)| = |*ρ*_↑_(**q**, *ω*)|*_mag_* − |*ρ*_↑_(**q**, *ω*)|*_nonmag_*, which can be viewed as the magnetic-field-induced weight transfer as shown in Fig. 3(A) of Ref. 21 and Fig. 4 of Ref. 22. As we can see, the behaviors of the QPI vectors indeed meet our expectations, that is, the sign-preserving and sign-reversing **q***_i_* are enhanced and suppressed by the introduction of vortices, respectively.

As to the Zeeman splitting, it is expected to be *gμ_B_B* ~ 0.9 meV at *B* = 8T (*g* = 2). Supposing the SC gap in Sr_2_IrO_4_ to be Δ ~ 9 meV, then the Zeeman energy is estimated to be 0.1Δ. In this case, the chemical potentials in equation (3) for the spin up and down electrons differ by this Zeeman energy^25^ and we again calculate the QPI spectra (not shown here). We found that, at this value of the Zeeman energy, the main effects are a tiny splitting of the CECs and a slight displacement of the QPI vectors. In the meantime, the intensity of the QPI vectors stays almost unchanged. Therefore, experimentally the effect of the Zeeman splitting can be neglected.

Then we explain the reason why we adopt a scattering matrix Ω that is diagonal in the orbital basis. As we know, in single-band superconductors like the cuprates, the intensity of the QPI vectors is solely determined by the coherence factor *C*(**k***_i_*, **k***_f_*), which is a combination of the BCS coefficients *u_k_* and *v_k_*. On the contrary, in multi-orbital superconductors, the intensity of the QPI vectors is determined not only by the coherence factor, but also by the matrix elements of the unitary transformation between the orbital and band bases. This can be seen from equation (7), where the above mentioned two factors are both incorporated in the expression of *g*_0_(**k**, *ω*). Therefore, first of all, although we considered an impurity scattering matrix which is diagonal in the orbital basis [that is, we ignore the orbital effects in *T*(*ω*)], the orbital effects still affect the QPI spectra through *g*_0_(**k**, *ω*). Secondly, the orbital effects sometimes may blur the QPI vectors. For example, at *n* = 5.2, from the CEC shown in Fig. 1(b), if we neglect all the orbital effects, then the most pronounced QPI vectors should be 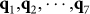 since the tip to tip scatterings generally have the largest joint density of states. Of course, the intensity of these QPI vectors are influenced by the coherence factor *C*(**k***_i_*, **k***_f_*). However, if the orbital effects are taken into account, then the joint density of states associated with the tip to tip scatterings is affected by the orbital to band transformation (*i.e.*, the variation of the orbital content along the CECs) and the intensity of the QPI vectors is influenced not only by *C*(**k***_i_*, **k***_f_*), but also by this transformation and this may be the reason why **q**_4_ and **q**_5_ cannot be clearly seen. In this case, if we further consider the orbital effect of the impurity scattering [that is, we add some off-diagonal elements in equation (4)], then the QPI vectors may be further blurred. Thirdly, to the best of our knowledge, in multi-orbital systems, the exact orbital effects of the impurity scattering have not been determined, either experimentally or from first principles calculations. Therefore, in our work, we made the simplest approximation and predicted some QPI vectors which can be observed in experiments in the ideal case.

At last, we would like to mention that we have also calculated the spectra for *Z*_↑_(**q**, *ω*), which is the Fourier transform of *Z*_↑_(**r**, *ω*) = *ρ*_↑_(**r**, *ω*)/*ρ*_↑_(**r**, −*ω*). Experimentally this procedure can eliminate extrinsic effects associated with the scanning feedback loop^21^,^22^ and suppress the checkerboard signal^21^. However in our theoretical investigation, the above two factors do not exist and the spectra for *Z*_↑_(**q**, *ω*) are qualitatively the same as those for *ρ*_↑_(**q**, *ω*). Therefore the spectra for *Z*_↑_(**q**, *ω*) are not shown here.

In summary, we have studied the QPI spectra in both electron- and hole-doped Sr_2_IrO_4_, by assuming the pairing symmetries proposed by Refs. 15 and 16. In the electron-doped case, we found that the pairing functions in Refs. 15 and 16 are qualitatively the same and the QPI spectra can be explained based on a model similar to the octet model of the cuprates. On the other hand, for hole-doped Sr_2_IrO_4_, the QPI spectra in the SC phase resemble those of the iron pnictides where the interpocket scattering between the electron and hole pockets leads to a QPI vector locating at the nesting vector of these two pockets. In both cases, the evolution of the QPI vectors and their different behaviors in the nonmagnetic and magnetic impurity scattering cases can well be explained based on the evolution of the CEC and the sign structure of the SC order parameter. The QPI spectra presented in this paper can thus be compared with future STM experiments to test whether there are SC phases in electron- and hole-doped Sr_2_IrO_4_ and what the SC pairing symmetry is.

## Author Contributions

Y.G. supervised the whole work, performed the numerical calculations and analyzed the data. T.Z., H.X.H. and Q.H.W. joined in the data analysis. All of the authors contributed to the data interpretation and the writing of the manuscript.
